# Sex Hormone‐Based Models Outperform Conventional Scores for Metabolic Dysfunction‐Associated Steatotic Liver Disease Screening: A Sex‐Specific Analysis in Type 2 Diabetes

**DOI:** 10.1155/jdr/2030936

**Published:** 2026-10-07

**Authors:** Guixin Li, Weiwei Liu, Ruihua Chen, Jinghui Guo, Long Wang

**Affiliations:** ^1^ Department of Gastroenterology Shanghai Sixth People′s Hospital Affiliated to Shanghai Jiao Tong University School of Medicine Shanghai China; ^2^ Department of General Practice Sijing Town Community Healthcare Center Shanghai China; ^3^ The Metabolic Diseases Biobank Shanghai Sixth People′s Hospital Affiliated to Shanghai Jiao Tong University School of Medicine Shanghai China

**Keywords:** cardiovascular risk, estradiol, metabolic-associated steatotic liver disease, noninvasive screening, sex differences, testosterone, type 2 diabetes mellitus

## Abstract

**Background:**

Metabolic dysfunction‐associated steatotic liver disease (MASLD) is highly prevalent among patients with Type 2 diabetes mellitus (T2DM). Although various noninvasive assessment tools for MASLD are currently available, few have been specifically validated in T2DM populations. Furthermore, the role of sex hormones in MASLD and their potential influence on cardiovascular and renal outcomes remain incompletely understood.

**Methods:**

In this cross‐sectional study of 731 Chinese patients with T2DM, we evaluated associations of testosterone and estradiol with MASLD, cardiovascular risk, and renal function. Sex hormone‐based logistic regression models were constructed and compared with established noninvasive MASLD scores to assess their discriminative performance.

**Results:**

In male patients, higher testosterone, T/E2 ratio, FSH, and LH levels were inversely associated with MASLD, whereas higher DHEA levels were associated with an increased likelihood of MASLD. In female patients, higher DHEA levels were positively associated with MASLD, whereas prolactin showed a modest inverse association. After multivariable adjustment, testosterone remained independently and inversely associated with MASLD in males. Sex hormone‐based models demonstrated better discriminative performance than conventional noninvasive scoring systems for MASLD. Additionally, testosterone was inversely associated with the ApoB/ApoA1 ratio in males, whereas estradiol was positively associated with eGFR in females.

**Conclusion:**

In patients with T2DM, testosterone and estradiol showed sex‐specific associations with metabolic outcomes. Sex hormone‐based models may provide a useful noninvasive approach for MASLD screening and risk assessment in T2DM patients.

## 1. Introduction

Metabolic dysfunction‐associated steatotic liver disease (MASLD) is diagnosed based on the presence of hepatic steatosis confirmed by imaging or liver biopsy, in conjunction with at least one cardiometabolic risk factor [1, 2]. The global prevalence of MASLD (formerly known as NAFLD) is estimated to be approximately 30%–40%, with a similar prevalence of 29.6%–31.6% reported in Asian populations across meta‐analyses [1, 3, 4].

Type 2 diabetes mellitus (T2DM) and MASLD are bidirectionally interconnected, each condition influencing the development and progression of the other. MASLD affects up to 70% of individuals with T2DM, and metabolic dysfunction‐associated steatohepatitis (MASH) occurs in as many as 37% of these patients, compared with approximately 7% in the general population [5]. Furthermore, T2DM is associated with an increased risk of disease progression from simple steatosis to advanced fibrosis, cirrhosis, hepatocellular carcinoma (HCC), and other adverse liver‐related outcomes [6]. Conversely, approximately 23% of individuals with MASLD have T2DM, and MASLD is closely linked to broader metabolic dysfunction, including an elevated risk of cardiovascular events [5, 7]. The growing burden of MASLD coexisting with T2DM underscores the urgent need to identify drivers of disease progression and develop noninvasive strategies for early detection and risk stratification.

Accordingly, current guidelines recommend ultrasonography, liver elastography, and magnetic resonance imaging (MRI) for screening hepatic steatosis in individuals with T2DM, given their substantially increased risk of adverse liver outcomes [1]. However, the limited accessibility and relatively high cost may restrict their widespread implementation, particularly in primary healthcare settings. Serum‐based noninvasive biomarkers thus represent a practical and cost‐effective alternative for large‐scale MASLD screening. While indices such as the aspartate aminotransferase (AST)/alanine aminotransferase (ALT) ratio, FIB‐4, and APRI are recommended for fibrosis assessment and identification of individuals at high risk of MASH, their performance in detecting early hepatic steatosis remains uncertain. Moreover, other widely used indices in MASLD research, including the fatty liver index (FLI), hepatic steatosis index (HSI), lipid accumulation product (LAP), and visceral adiposity index (VAI), still require further validation for steatosis screening purposes [8, 9].

Notably, MASLD exhibits distinct sex‐related patterns, with a higher prevalence and earlier onset observed in men than in women [10]. Despite an approximately 20% lower overall risk of MASLD and no significant sex difference in MASH risk, women have a 37% higher risk of advanced fibrosis than men, highlighting sex‐specific differences in fibrotic outcomes [10]. Furthermore, MASLD is associated with a significantly higher risk of cardiovascular disease events and certain cancers in women than in men [10–12]. In addition, MASLD is independently associated with an increased risk of chronic kidney disease (CKD) and HCC, with limited evidence indicating a stronger association in men than in women [10, 11]. These sex‐related differences may be partly mediated by sex hormones, which regulate MASLD through effects on lipid metabolism, mitochondrial function, oxidative stress, immune responses, and tissue repair [13]. Importantly, despite sex‐related differences in MASLD susceptibility, commonly used noninvasive tools such as controlled attenuation parameter (CAP), FLI, and HSI generally apply uniform thresholds across sexes, which may reduce diagnostic accuracy [11, 13]. Additionally, variations in metabolic profiles between Asian and Western populations may restrict the applicability of existing prediction models in specific ethnic populations [14]. These limitations highlight the need for tailored approaches in Chinese patients with T2DM that consider sex‐specific characteristics and population‐specific contexts.

Given the established sex‐related differences in MASLD prevalence, progression, and extrahepatic outcomes, and the potential role of sex hormones in metabolic homeostasis, we hypothesized that sex hormones may contribute to sex‐specific differences in MASLD among patients with T2DM and could facilitate better identification of at‐risk individuals. Accordingly, this study is aimed at (1) investigating the associations between sex hormones and MASLD in Chinese patients with T2DM, stratified by sexes; (2) evaluating whether sex hormone‐based models could improve the diagnostic performance of current noninvasive MASLD assessment tools; and (3) exploring the associations of sex hormones with ischemic cardiovascular disease (ICVD) risk and renal function, to assess their potential clinical relevance in metabolic‐related diseases.

## 2. Methods

### 2.1. Study Participants

This study was conducted at Shanghai Sixth People′s Hospital and included adult patients (≥ 18 years old) diagnosed with T2DM for whom complete clinical data were available between January 2021 and December 2022.

Patients were excluded based on the following criteria: (1) chronic alcohol consumption, defined as ethanol intake ≥ 20 g/day or ≥ 140 g/week for men, and ≥ 10 g/day or ≥ 70 g/week for women; (2) presence of viral hepatitis (including positive hepatitis B surface antigen or anti‐hepatitis C antibody), drug‐induced liver injury, biliary diseases, cirrhosis, Wilson′s disease, autoimmune liver diseases, hemochromatosis, or *α*‐1 antitrypsin deficiency; (3) use of hepatotoxic medications (e.g., glucocorticoids, amiodarone, tamoxifen, valproic acid, thiotepa, tetracycline, or methotrexate) within the preceding 6 months; (4) endocrine disorders, including hyperthyroidism, hypothyroidism, Cushing′s syndrome, pheochromocytoma, pituitary tumors, or anterior pituitary insufficiency; and (5) women with a history of bilateral oophorectomy and hysterectomy, or prior breast or gynecological malignancies.

The study protocol was approved by the Ethics Committee of Shanghai Sixth People′s Hospital (Approval No. 2020‐069), and written informed consent was obtained from all participants prior to enrollment.

### 2.2. Diagnosis of MASLD and Noninvasive Assessment Indices

MASLD was diagnosed based on ultrasonography findings, which were independently reviewed by experienced ultrasound specialists blinded to clinical and laboratory data according to established imaging criteria. Hepatic steatosis was identified by characteristic features, including increased hepatic echogenicity, reduced penetration of the ultrasound beam, and decreased visualization of intrahepatic vessels and deeper structures [15].

Noninvasive assessment indices were derived from commonly available biochemical markers, including fasting insulin (mIU/L), fasting plasma glucose (FPG, mmol/L), age (years), AST (U/L), ALT (U/L), platelet count (10^9^/L), testosterone (nmol/L), estradiol (pmol/L), body mass index (BMI, kg/m^2^), waist circumference (cm), triglycerides (TG, mmol/L), *γ*‐glutamyl transferase (GGT, U/L), high‐density lipoprotein cholesterol (HDL‐C, mmol/L), and serum creatinine (Scr, *μ*mol/L). Blood samples for all laboratory measurements were collected in the morning after an overnight fast to minimize potential diurnal variation, particularly for sex hormones.

The homeostasis model assessment of insulin resistance (HOMA‐IR) was calculated using the following formula [8]:

(1) HOMA−IR=Fasting insulin×FPG22.5

The acMASH index was calculated using the formula [16]

(2) acMASH=ASTScr×10

acMASH index <4.15 was used to rule out MASH, whereas acMASH index ≥ 4.15 indicated a higher risk of MASH.

The FIB‐4 index was calculated using the formula [9]

(3) FIB−4=Age×ASTPlatelet count×ALT

FIB‐4 ≥ 1.3 indicated a higher risk of advanced fibrosis.

The T/E2 ratio was calculated using the formula [17]

(4) T/E2 ratio=TestosteroneEstradiol×0.0943

FLI was calculated using the formula [9]

(5) FLI=e0.9530.1390.7180.05315.745×ln88.54×TG+×BMI+×lnGGT+×Waist circumference−1+e0.9530.1390.7180.05315.745×ln88.54×TG+×BMI+×lnGGT+×Waist circumference−×100

FLI ≥ 60 indicated a high probability of fatty liver.

HSI was calculated using the formula [9]

(6) HSI=ALT/AST×82+BMI+Male HSI=ALT/AST×822+BMI++ Female

HSI > 36 suggested the presence of fatty liver.

LAP was calculated using the formula [8]

(7) LAP=Waist circumference−65×TG MaleLAP=Waist circumference−58×TG Female

VAI was calculated using the formula [8]

(8) VAI=Waist circumference39.681.88+×BMI×TG1.03×1.31HDL−CMaleVAI=Waist circumference36.581.89+×BMI×TG0.81×1.52HDL−CFemale

ICVD events were defined as coronary heart disease and ischemic stroke. The 10‐year risk for patients aged 35–59 years was estimated using a simplified scoring system [18]. Participants within this age range were extracted from our cohort and stratified into five risk categories based on the calculated ICVD scores: very low (< 1%), low (1%–5%), moderate (5.1%–10%), high (10.1%–20%), and very high (> 20%).

The chronic kidney disease epidemiology collaboration (CKD‐EPI) equation was used to estimate renal function, as described below:

(9) eGFR=141×min0.0113×Scr0.9,1−0.411×max0.0113×Scr0.9,1−1.209×0.993AgeMaleeGFR=1411.018×min0.0113×Scr0.7,1−0.329×max0.0113×Scr0.7,1−1.209×0.993Age× Female

### 2.3. Statistical Analysis

The primary analyses assessed the associations of testosterone and estradiol with MASLD using multivariable logistic regression. Correlation and univariable logistic regression analyses were considered exploratory. No adjustment for multiple comparisons was performed for these exploratory analyses, and their findings were interpreted as hypothesis‐generating. All statistical analyses were performed using PASW Statistics 18 (SPSS Inc., United States) and R Studio (Posit, United States).

Missing data were minimal: one value (0.2%) was missing in the MASLD with T2DM group, and two values (0.7%) were missing in the T2DM‐only group. Given the low proportion of missing data and the assumption that they were missing completely at random, we excluded the missing values through complete case analysis for all subsequent analyses.

Normality of continuous variables were assessed using the Shapiro–Wilk test. Since all continuous variables were found to be non‐normally distributed, they are presented as median with interquartile range (IQR). Comparisons between two groups were performed using the Mann–Whitney *U* test, whereas comparisons among more than two groups were conducted using the Kruskal–Wallis test. Categorical variables were presented as weighted proportions with 95% confidence intervals (CI) where appropriate, and comparisons were performed using the *χ* ^2^ test.

Spearman′s rank correlation was used to assess associations between variables. To adjust for the potential confounding effect of age, partial correlation analyses were performed on ranked data.

Multicollinearity was assessed using variance inflation factors (VIF), and no significant multicollinearity was detected (all VIFs < 5). Subsequently, binary logistic regression was performed to evaluate risk factors, with results presented as odds ratios (ORs) and 95% CIs.

Restricted cubic spline (RCS) regression with four knots was applied to examine potential nonlinear associations between sex hormone levels and MASLD.

The discriminative performance of sex hormone‐based models for MASLD was evaluated using receiver operating characteristic (ROC) curve analysis, with discrimination quantified by the area under the curve (AUC). The DeLong test was used to compare the AUCs of different models and to determine whether the differences in diagnostic performance were statistically significant. Internal validation was performed using 1000 bootstrap resamples. For each resample, the sex‐specific logistic regression model was refitted, and model optimism was estimated from the difference in AUC between the bootstrap and original datasets. The optimism‐corrected AUC was calculated by subtracting the mean optimism from the apparent AUC.

All statistical tests were two‐tailed, and *p* < 0.05 was considered statistically significant.

## 3. Results

### 3.1. Baseline Characteristics of the Study Population

A total of 731 adults with T2DM were included in the study, with a median age of 61 years (IQR, 52–68 years). The cohort comprised 489 males (66.9%) and 242 females (33.1%), of whom 195 (80.6%) were postmenopausal. MASLD was diagnosed in 427 participants (58.4%). Baseline characteristics stratified by MASLD status are summarized in Table 1.

**Table 1:** Baseline characteristics of the study population.

	T2DM only (*n* = 304)	MASLD with T2DM (*n* = 427)	*p* value
Age (years)	63 (57~70)	59 (48~66)	**< 0.001**
Sex (female%)	32.6 (27.3, 37.9)	33.5 (29.0, 38.0)	0.794
BMI (kg/m^2^)	22.81 (20.98~24.75)	25.61 (23.75~27.68)	**< 0.001**
Waist circumference (cm)	88.0 (88.5~94.0)	95.0 (90~101)	**< 0.001**
WHR	0.93 (0.88~0.97)	0.96 (0.92~1.00)	**< 0.001**
Hypertension (%)	42.8 (37.2, 48.4)	49.4 (44.7, 54.2)	0.076
Smoking (%)	32.9 (27.6, 38.2)	34.7 (30.1, 39.2)	0.619
Total cholesterol (mmol/L)	4.63 (3.94~5.46)	4.84 (4.18~5.63)	**0.002**
Triglyceride (mmol/L)	1.02 (0.74~1.48)	1.60 (1.15~2.44)	**< 0.001**
HDL‐C (mmol/L)	1.15 (0.94~1.37)	1.00 (0.85~1.18)	**< 0.001**
LDL‐C (mmol/L)	2.61 (2.14~3.32)	2.93 (2.34~3.52)	**< 0.001**
FPG (mmol/L)	6.43 (5.31~7.80)	7.11 (5.91~8.58)	**< 0.001**
HbA1c (%)	8.35 (7.00~9.95)	8.80 (7.50~10.10)	**0.013**
PLT (10^9^/L)	220.0 (180.0~255.5)	225.0 (193.5~265.0)	**0.019**
ALT (U/L)	16.0 (13.0~22.0)	24.0 (18.0~37.5)	**< 0.001**
AST (U/L)	18.0 (15.0~21.0)	20.0 (17.0~26.5)	**< 0.001**
ALP (U/L)	74.0 (58.0~88.0)	73.0 (61.0~87.0)	0.635
GGT (U/L)	18.0 (13.0~27.0)	25.0 (18.0~39.5)	**< 0.001**
Scr (*μ*mol/L)	70.45 (61.55~81.95)	72.10 (62.40~84.30)	0.523
BUN (mmol/L)	6.1 (5.1~7.5)	5.8 (4.9~6.8)	**0.002**
HOMA‐IR	2.81 (1.39~5.05)	4.31 (2.52~6.91)	**< 0.001**
acMASH	2.55 (2.02~3.25)	2.86 (2.17~3.98)	**< 0.001**
FIB‐4	1.28 (0.97~1.72)	1.08 (0.78~1.48)	**< 0.001**
FLI	98.5 (98.0~99.0)	99.1 (98.7~99.4)	**< 0.001**
HSI	33.0 (30.9~35.8)	38.4 (35.4~41.7)	**< 0.001**
LAP	25.7 (15.1~40.9)	51.8 (35.5~81.2)	**< 0.001**
VAI	1.43 (0.88~2.16)	2.69 (1.64~4.13)	**< 0.001**
Male (*n* = 489)			
Testosterone (nmol/L)	13.40 (10.60~17.00)	10.92 (7.85~14.15)	**< 0.001**
Estradiol (pmol/L)	79.60 (53.90~103.00)	82.09 (60.95~104.50)	0.216
T/E2	1.81 (1.35~2.68)	1.39 (1.02~1.96)	**< 0.001**
FSH (IU/L)	8.89 (5.85~12.70)	7.45 (4.84~11.45)	**0.004**
LH (IU/L)	7.75 (5.38~10.62)	6.62 (5.03~9.06)	**0.012**
DHEA (*μ*g/dL)	180.0 (128.0~253.0)	212.0 (142.5~295.5)	**0.005**
Progesterone (nmol/L)	0.21 (0.16~0.36)	0.19 (0.16~0.36)	0.620
Prolactin (mIU/L)	181.0 (145.2~235.0)	188.0 (142.7~236.5)	0.791
Female (*n* = 242)			
Testosterone (nmol/L)	0.24 (0.09~0.39)	0.34 (0.13~0.57)	**0.021**
Estradiol (pmol/L)	21.20 (18.40~42.90)	27.30 (18.40~58.20)	0.653
T/E2	0.07 (0.04~0.15)	0.08 (0.05~0.19)	0.250
FSH (IU/L)	48.60 (34.98~64.71)	44.17 (29.33~61.52)	0.216
LH (IU/L)	24.60 (17.93~30.60)	22.44 (14.63~31.98)	0.257
DHEA (*μ*g/dL)	107.0 (73.9~155.6)	120.5 (83.7~189.0)	0.060
Progesterone (nmol/L)	0.16 (0.16~0.36)	0.16 (0.16~0.34)	0.777
Prolactin (mIU/L)	214.0 (171.0~327.0)	194.4 (147.5~268.6)	**0.019**
Premenopausal women (*n* = 47)			
Testosterone (nmol/L)	0.33 (0.14~0.39)	0.49 (0.20~0.68)	0.084
Estradiol (pmol/L)	102.10 (29.80~226.90)	141.00 (68.67~273.70)	0.302
T/E2	0.03 (0.01~0.12)	0.02 (0.02~0.06)	0.974
FSH (IU/L)	5.99 (3.95~46.57)	6.67 (4.80~10.10)	0.584
LH (IU/L)	9.94 (3.80~21.00)	7.00 (5.63~9.68)	0.770
DHEA (*μ*g/dL)	111.0 (76.0~180.0)	138.0 (113.0~225.0)	0.071
Progesterone (nmol/L)	0.23 (0.16~7.14)	0.37 (0.16~7.30)	0.301
Prolactin (mIU/L)	291.5 (208.9~495.7)	263.0 (195.0~348.6)	0.325
Postmenopausal women (*n* = 195)			
Testosterone (nmol/L)	0.23 (0.09~0.39)	0.29 (0.12~0.54)	0.064
Estradiol (pmol/L)	18.40 (18.40~38.10)	20.51 (18.40~37.20)	0.867
T/E2	0.08 (0.05~0.17)	0.10 (0.05~0.21)	0.093
FSH (IU/L)	53.88 (38.00~65.30)	48.76 (35.21~64.80)	0.480
LH (IU/L)	26.30 (21.30~33.12)	24.63 (17.60~33.20)	0.326
DHEA (*μ*g/dL)	104.0 (70.7~149.8)	108.6 (81.7~178.0)	0.235
Progesterone (nmol/L)	0.16 (0.16~0.32)	0.16 (0.16~0.27)	0.692
Prolactin (mIU/L)	210.8 (165.0~287.0)	185.2 (145.0~242.0)	**0.019**

*Note:* Data are shown as median (interquartile range) or weighted proportion (95% confidence interval). Bold data indicate statistically significant differences between groups (p < 0.05).

Abbreviations: ALP, alkaline phosphatase; ALT, alanine aminotransferase; AST, aspartate aminotransferase; BMI, body mass index; BUN, blood urea nitrogen; DHEA, dehydroepiandrosterone; FLI, fatty liver index; FPG, fasting plasma glucose; FSH, follicle‐stimulating hormone; GGT, gamma glutamyltransferase; HbA1c, hemoglobin A1c; HDL‐C, high‐density lipoprotein‐cholesterol; HSI, hepatic steatosis index; LAP, lipid accumulation product; LDL‐C, low‐density lipoprotein‐cholesterol; LH, luteinizing hormone; PLT, platelet; Scr, serum creatinine; T/E2, testosterone‐to‐estradiol ratio; VAI, visceral adiposity index; WHR, waist–hip ratio.

Compared with patients with T2DM alone, those with coexisting MASLD presented with a more unfavorable metabolic profile, characterized by greater adiposity, impaired glucose and lipid metabolism, elevated liver enzymes, increased insulin resistance, and higher MASLD‐related noninvasive indices. In contrast, age, HDL‐C, blood urea nitrogen (BUN), and FIB‐4 index were lower in patients with MASLD.

Regarding sex hormones, male patients with MASLD exhibited lower testosterone, T/E2 ratio, follicle‐stimulating hormone (FSH), and luteinizing hormone (LH) levels, along with higher dehydroepiandrosterone (DHEA) levels, compared to those without MASLD. In female patients, those with MASLD had higher testosterone and lower prolactin levels than their counterparts without MASLD. Stratified analyses by menopausal status showed that, among postmenopausal women, prolactin was the only hormone exhibiting a significant difference between individuals with and without MASLD.

### 3.2. Correlation of Sex Hormones With Metabolic Parameters in T2DM Patients

Given the sex‐specific differences in hormone regulation and metabolism, correlations between sex hormones and metabolic parameters were analyzed separately for males and females (Tables 2 and 3).

**Table 2:** Correlation of sex hormone levels with metabolic as well as hepatic parameters in male T2DM patients.

	Testosterone				Estradiol				T/E2			
	*r*	*p* value	Age‐adjusted		*r*	*p* value	Age‐adjusted		*r*	*p* value	Age‐adjusted	
			*r*	*p* value			*r*	*p* value			*r*	*p* value
Age	0.15	**0.001**	—	—	0.00	0.999	—	—	0.12	**0.008**	—	—
BMI	−0.35	**< 0.001**	−0.32	**< 0.001**	0.10	**0.023**	0.11	**0.012**	−0.39	**< 0.001**	−0.38	**< 0.001**
Waist circumference	−0.32	**< 0.001**	−0.30	**< 0.001**	0.15	**0.001**	0.15	**0.001**	−0.40	**< 0.001**	−0.40	**< 0.001**
WHR	−0.26	**< 0.001**	−0.25	**< 0.001**	0.12	**0.009**	0.12	**0.007**	−0.33	**< 0.001**	−0.33	**< 0.001**
Total cholesterol	−0.05	0.244	−0.02	0.615	−0.04	0.431	−0.04	0.416	−0.03	0.525	0.00	0.920
Triglyceride	−0.34	**< 0.001**	−0.32	**< 0.001**	0.01	0.752	0.01	0.747	−0.30	**< 0.001**	−0.28	**< 0.001**
HDL‐C	0.29	**< 0.001**	0.27	**< 0.001**	−0.03	0.545	−0.03	0.552	0.25	**< 0.001**	0.24	**< 0.001**
LDL‐C	−0.03	0.458	0.00	0.976	0.00	0.996	0.00	0.994	−0.05	0.277	−0.02	0.595
FPG	−0.25	**< 0.001**	−0.23	**< 0.001**	0.03	0.505	0.04	0.433	−0.21	**< 0.001**	−0.20	**< 0.001**
HbA1c	−0.04	0.358	−0.03	0.560	−0.01	0.879	−0.01	0.812	−0.04	0.326	−0.03	0.527
ALT	−0.26	**< 0.001**	−0.23	**< 0.001**	0.07	0.117	0.08	0.084	−0.30	**< 0.001**	−0.28	**< 0.001**
AST	−0.11	**0.016**	−0.09	**0.045**	0.13	**0.004**	0.13	**0.003**	−0.24	**< 0.001**	−0.23	**< 0.001**
ALP	−0.09	0.054	−0.08	0.067	−0.01	0.775	−0.02	0.722	−0.07	0.098	−0.07	0.134
GGT	−0.17	**< 0.001**	−0.13	**0.004**	0.11	**0.017**	0.12	**0.010**	−0.24	**< 0.001**	−0.22	**< 0.001**
HOMA‐IR	−0.20	**< 0.001**	−0.18	**< 0.001**	0.00	0.963	0.01	0.841	−0.16	**< 0.001**	−0.15	**0.001**

*Note:* Bold data indicate statistically significant differences between groups (p < 0.05).

Abbreviations: ALP, alkaline phosphatase; ALT, alanine aminotransferase; AST, aspartate aminotransferase; BMI, body mass index; FPG, fasting plasma glucose; HbA1c, hemoglobin A1c; HDL‐C, high‐density lipoprotein‐cholesterol; LDL‐C, low‐density lipoprotein‐cholesterol; PLT, platelet; T/E2, testosterone‐to‐estradiol ratio; WHR, waist‐hip ratio.

**Table 3:** Correlation of sex hormone levels with metabolic as well as hepatic parameters in female T2DM patients.

	Testosterone				Estradiol				T/E2			
	*r*	*p* value	Age‐adjusted		*r*	*p* value	Age‐adjusted		*r*	*p* value	Age‐adjusted	
			*r*	*p* value			*r*	*p* value			*r*	*p* value
Age	−0.11	0.094	—	—	−0.31	**< 0.001**	—	—	0.26	**< 0.001**	—	—
BMI	0.22	**< 0.001**	0.21	**0.001**	0.22	**0.001**	0.19	**0.003**	0.00	0.966	0.04	0.537
Waist circumference	0.17	**0.010**	0.17	**0.010**	0.16	**0.010**	0.17	**0.008**	−0.01	0.890	−0.01	0.905
WHR	0.03	0.645	0.04	0.566	0.02	0.750	0.04	0.508	−0.04	0.580	−0.06	0.393
Total cholesterol	−0.04	0.558	−0.05	0.452	0.06	0.370	0.03	0.643	−0.12	0.064	−0.10	0.131
Triglyceride	0.08	0.190	0.06	0.327	0.14	**0.035**	0.08	0.241	−0.05	0.432	0.01	0.927
HDL‐C	−0.12	0.070	−0.10	0.107	−0.13	0.052	−0.09	0.163	−0.06	0.353	−0.10	0.126
LDL‐C	−0.02	0.746	−0.03	0.665	0.03	0.626	0.01	0.848	−0.07	0.254	−0.06	0.363
FPG	0.05	0.477	0.03	0.668	0.06	0.369	0.01	0.936	−0.05	0.457	0.00	0.971
HbA1c	−0.04	0.506	−0.04	0.512	−0.02	0.795	−0.02	0.813	−0.03	0.641	−0.03	0.609
ALT	0.12	0.054	0.11	0.100	0.15	**0.018**	0.10	0.117	−0.03	0.621	0.02	0.769
AST	0.04	0.569	0.03	0.633	0.08	0.206	0.07	0.297	−0.02	0.704	−0.01	0.878
ALP	−0.12	0.069	−0.10	0.126	−0.12	0.055	−0.07	0.284	0.00	0.989	−0.05	0.414
GGT	0.14	**0.032**	0.13	**0.040**	0.10	0.129	0.08	0.194	0.02	0.755	0.04	0.566
HOMA‐IR	0.09	0.154	0.08	0.196	0.20	**0.002**	0.18	**0.005**	−0.07	0.280	−0.05	0.448

*Note:* Bold data indicate statistically significant differences between groups (p < 0.05).

Abbreviations: ALP, alkaline phosphatase; ALT, alanine aminotransferase; AST, aspartate aminotransferase; BMI, body mass index; FPG, fasting plasma glucose; GGT, gamma glutamyltransferase; HbA1c, hemoglobin A1c; HDL‐C, high‐density lipoprotein‐cholesterol; LDL‐C, low‐density lipoprotein‐cholesterol; PLT, platelet; T/E2, testosterone‐to‐estradiol ratio; WHR, waist–hip ratio.

In male patients, age‐adjusted analyses revealed that lower testosterone levels were consistently associated with greater adiposity, dyslipidemia, impaired glycemic control, elevated liver enzymes, and increased insulin resistance. Conversely, testosterone was positively associated with HDL‐C. Estradiol showed positive associations with several adiposity‐ and liver‐related parameters, whereas the T/E2 ratio exhibited a correlation pattern similar to that of testosterone.

In female patients, testosterone was positively associated with adiposity and GGT, whereas estradiol was positively associated with adiposity and insulin resistance. No significant correlations were observed between the T/E2 ratio and metabolic or hepatic parameters.

In exploratory analyses of liver steatosis and fibrosis parameters, testosterone was inversely associated with both CAP and LSM in males, whereas progesterone and DHEA showed positive associations with CAP and LSM, respectively. In females, estradiol and progesterone were positively associated with LSM (Table S1).

### 3.3. Univariate and Multivariate Logistic Regression Analysis for MASLD in T2DM Patients

Univariate logistic regression analysis identified several sex hormones associated with MASLD (Table S2, Figure 1). In male patients, higher testosterone, T/E2 ratio, FSH, and LH levels were associated with a decreased risk of MASLD, whereas higher DHEA levels were associated with an increased risk (Figure 1A). In female patients, only higher DHEA and lower prolactin levels were significantly associated with MASLD (Figure 1B). Among postmenopausal women, prolactin remained the only hormone significantly associated with MASLD. Subgroup analyses stratified by acMASH and FIB‐4 consistently showed an inverse association between testosterone and MASLD in male patients (Tables S3 and S4).

**Figure 1:**
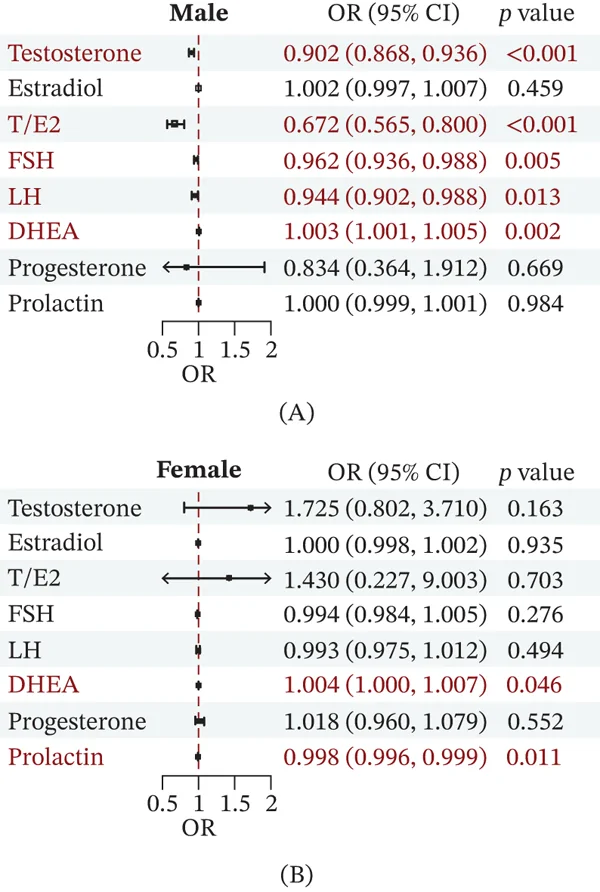
Univariate logistic regression analysis for MASLD in (A) male and (B) female T2DM patients.

Multivariable logistic regression analyses were subsequently performed in males, females, and postmenopausal women (Table 4). In male patients, testosterone remained independently associated with lower odds of MASLD after adjustment for potential confounders (OR [95% CI]: 0.952 [0.907, 0.999]), whereas higher BMI, TC, and ALT were independently associated with increased odds. In female and postmenopausal patients, BMI and ALT remained independent risk factors, whereas HDL‐C was independently associated with lower odds of MASLD.

**Table 4:** Multivariate logistic regression analysis for MASLD in T2DM patients.

	Male		Female		Postmenopausal women	
	OR (95% CI)	*p* value	OR (95% CI)	*p* value	OR (95% CI)	*p* value
Age	0.978 (0.957, 0.999)	**0.044**	1.001 (0.967, 1.036)	0.970	0.989 (0.941, 1.039)	0.653
Age at menopause	—	—	—	—	0.913 (0.822, 1.014)	0.089
BMI	1.367 (1.241, 1.506)	**< 0.001**	1.245 (1.117, 1.387)	**< 0.001**	1.242 (1.105, 1.397)	**< 0.001**
Smoking	1.429 (0.911, 2.240)	0.120	4.844 (0.447, 52.440)	0.194	4.674 (0.347, 62.877)	0.245
Hypertension	1.018 (0.635, 1.634)	0.940	0.977 (0.497, 1.921)	0.946	0.870 (0.416, 1.817)	0.711
TC	1.268 (1.010, 1.592)	**0.041**	1.199 (0.916, 1.568)	0.186	1.190 (0.888, 1.596)	0.244
HDL‐C	0.487 (0.192, 1.236)	0.130	0.139 (0.049, 0.395)	**< 0.001**	0.164 (0.054, 0.500)	**0.001**
HbA1c	1.082 (0.968, 1.211)	0.166	0.957 (0.804, 1.138)	0.618	0.929 (0.769, 1.123)	0.448
ALT	1.030 (1.012, 1.047)	**0.001**	1.043 (1.018, 1.069)	**0.001**	1.041 (1.011, 1.071)	**0.008**
Testosterone	0.952 (0.907, 0.999)	**0.047**	1.186 (0.454, 3.099)	0.727	1.015 (0.350, 2.940)	0.978
Estradiol	1.002 (0.996, 1.009)	0.540	0.998 (0.995, 1.001)	0.120	0.997 (0.993, 1.001)	0.093

*Note:* Bold data indicate statistically significant differences between groups (p < 0.05).

Abbreviations: ALT, alanine aminotransferase; BMI, body mass index; HbA1c, hemoglobin A1c; HDL‐C, high‐density lipoprotein‐cholesterol.

### 3.4. RCS Analysis of Testosterone and MASLD in Male T2DM Patients

Given the consistent inverse association between testosterone and MASLD observed in the regression analyses, RCS analysis was performed to further characterize this relationship. The RCS analysis supported an overall inverse association between testosterone and MASLD, with no evidence of significant departure from linearity (*p* overall < 0.0001; *p* nonlinear = 0.252) (Figure 2).

**Figure 2:**
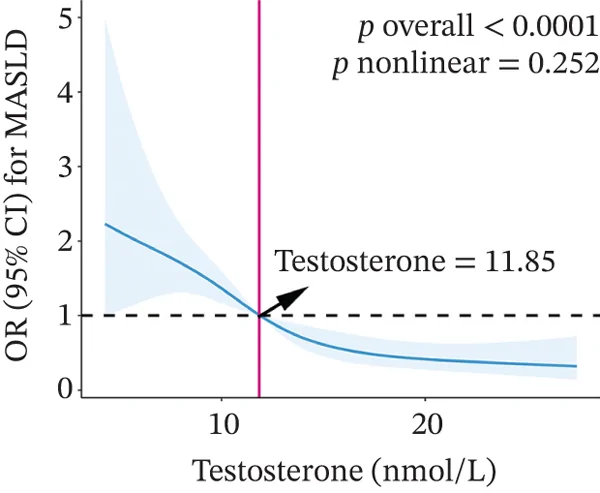
Restricted cubic spline analysis of testosterone and MASLD in the male T2DM patients.

### 3.5. Predictive Performance of Serum Sex Hormones, Sex Hormone‐Based Models, and Noninvasive Assessment Tools for MASLD in T2DM Patients

The predictive performance of serum sex hormones, sex hormone‐based models, and noninvasive assessment tools for MASLD was evaluated using ROC curve analysis. In male patients, serum testosterone alone showed moderate discriminative ability for MASLD (Figure 3A). Incorporation of clinical variables and sex hormones improved the predictive performance of the model, with an apparent AUC of 0.820 (Figure 3B, Table S5). Compared with the sex hormone‐based model, FLI, HSI, and VAI demonstrated significantly lower diagnostic performance, whereas LAP was comparable (Figure 3C–F, Table S5).

**Figure 3:**
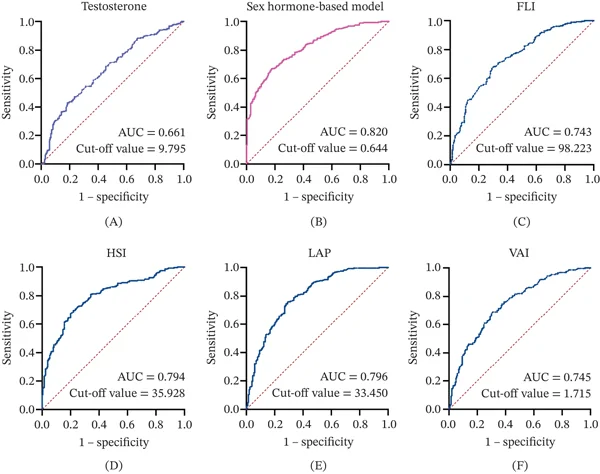
Receiver operating characteristic curves showing the predictive performance of (A) serum testosterone, (B) sex hormone‐based model, and (C–F) noninvasive assessment tools for MASLD in men with T2DM. Sex hormone‐based model for males: Logit (Probability of MASLD) = −7.466−0.022 × Age+0.313 × BMI +0.357 × Smoking (No: 0; Yes: 1) +0.018 × Hypertension (No: 0; Yes: 1) +0.237 × TC −0.719 × HDL‐C +0.079 × HbA1c +0.029 × ALT −0.049 × Testosterone +0.002 × Estradiol. Abbreviations: AUC, area under the curve; BMI, body mass index; HDL‐C, high‐density lipoprotein‐cholesterol; HbA1c, hemoglobin A1c; ALT, alanine aminotransferase; FLI, fatty liver index; HSI, hepatic steatosis index; LAP, lipid accumulation product; VAI, visceral adiposity index.

In female patients, the sex hormone‐based model also demonstrated favorable discriminative ability, with an apparent AUC of 0.810 (Table S6). Compared with this model, FLI and VAI showed significantly inferior performance, whereas HSI and LAP exhibited comparable diagnostic accuracy (Table S6).

After internal validation using 1,000 bootstrap resamples, the optimism‐corrected AUCs were 0.804 and 0.776 in males and females, respectively (Table S7). Bootstrap correction resulted in a modest reduction in the estimated discriminative performance in both sexes.

### 3.6. Association of Sex Hormone Levels With Cardiovascular and Renal Risk

Patients were stratified according to a 10‐year ICVD risk. Serum testosterone and estradiol levels did not differ significantly across the 10‐year ICVD risk strata in either male or female patients (Figure 4A,C). In male patients, testosterone showed a modest inverse association with the ApoB/ApoA1 ratio, whereas estradiol was not associated with this cardiovascular marker (Figure 4B,D). Regarding renal function, estradiol was positively associated with eGFR in female patients, whereas no significant associations between sex hormones and eGFR were observed in males (Figure 4E,F).

**Figure 4:**
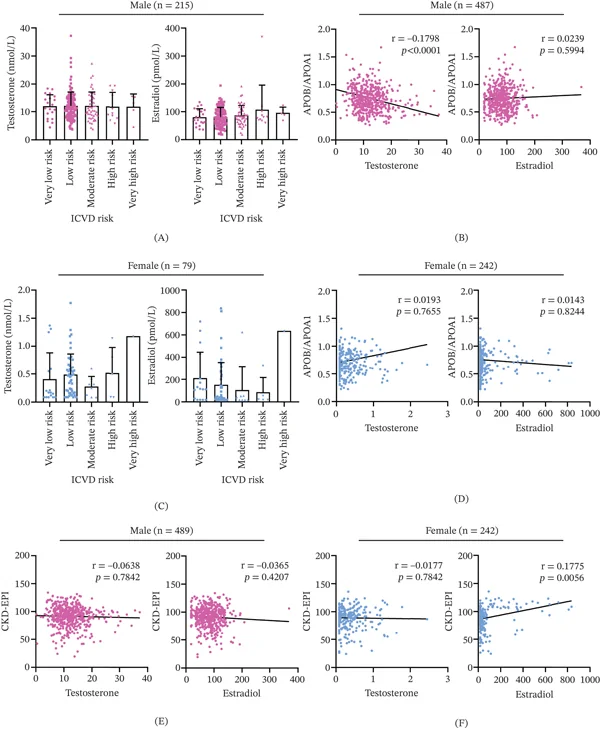
Association of sex hormone levels with cardiovascular and renal risk. (A, C) Serum testosterone and estradiol levels in (A) male and (C) female patients stratified by a 10‐year ICVD risk. Data are shown as mean ± SD. (B, D) Correlations between sex hormones and ApoB/ApoA1 ratio in (B) male and (D) female patients. (E,F) Correlations between sex hormones and eGFR calculated using the CKD‐EPI formula in (E) male and (F) female patients.

## 4. Discussion

In this study, patients with coexisting MASLD and T2DM were younger and had higher BMI and waist‐to‐hip ratio, reflecting greater central adiposity, compared with those with T2DM alone, although most were not obese (BMI ≤ 28 kg/m^2^). They also exhibited an adverse metabolic profile, characterized by dyslipidemia, hyperglycemia, and insulin resistance. According to noninvasive indices, only a small proportion of participants had acMASH values above the reported rule‐in threshold for MASH (acMASH > 7.73: 24/731) or FIB‐4 values suggestive of advanced liver fibrosis (FIB‐4 > 2.67: 25/731) [9, 16].

Nevertheless, ALT, AST, and GGT levels were higher in patients with MASLD, even though they remained within conventional laboratory reference ranges (ALT: 0–50 U/L, AST: 15–46 U/L, and GGT: 12–58 U/L). This observation aligns with previous reports indicating that individuals with MASLD may present with subtle liver enzyme elevations even when values fall within the normal range [19, 20]. However, serum liver enzymes have limited sensitivity and specificity for detecting MASLD, as a substantial proportion of affected individuals have normal ALT levels, whereas elevated liver enzyme may also arise from other hepatic or extrahepatic conditions [21]. Consequently, liver enzymes alone are insufficient for accurately identifying or excluding MASLD. Recent studies have further suggested that the conventional upper limits of normal for ALT may underestimate the risk of MASLD, prompting proposals for lower sex‐specific ALT thresholds (33 U/L for men and 25 U/L for women) and the integration of noninvasive assessment tools to enhance early detection and risk stratification of MASLD [1, 21].

Hepatic expression of over 1000 genes involved in steroid and lipid metabolism exhibits pronounced sex‐specific differences, most of which are driven by sex‐dependent growth hormone secretion patterns [22]. Importantly, these growth hormone‐related differences diminish after age 50, suggesting that postmenopausal women may experience substantial alterations in hepatic gene expression [22]. Accordingly, we performed stratified analyses for sex, with additional stratification of women by menopausal status. In male patients, higher circulating levels of testosterone, FSH, and LH were inversely associated with lower odds of MASLD, whereas higher prolactin levels were associated with lower odds of MASLD in females. In both sexes, higher DHEA levels were associated with higher odds of MASLD. Consistently, testosterone in men was negatively correlated with multiple metabolic abnormalities, independent of age. Moreover, higher testosterone levels remained associated with lower odds of MASLD in men after adjustment for potential confounders, consistent with previous reports [23, 24]. However, given the cross‐sectional nature of the present study, this association should not be interpreted as evidence of a protective effect or causal relationship. In particular, metabolic dysfunction and obesity may themselves influence circulating testosterone concentrations, raising the possibility of reverse causation. Although some studies have suggested that testosterone therapy may be associated with a lower risk of MASLD in male patients with T2DM [25–27], these findings also warrant caution, as some studies were not specifically designed to assess liver‐related outcomes, lacked comprehensive liver MRI assessment, or used conventional in‐phase/out‐of‐phase MRI sequences rather than MRI‐PDFF for hepatic fat quantification [25].

Notably, no significant association between estradiol levels and MASLD was observed in male or overall female T2DM patients. This finding contrasts with previous studies suggesting that estradiol exerts protective effects against MASLD through multiple mechanisms, including regulation of hepatic lipid metabolism, improvement of mitochondrial function, and attenuation of oxidative stress and inflammation [22]. These protective effects are more evident in premenopausal women, whereas the decline in estradiol levels after menopause is associated with increased susceptibility to MASLD [22]. We speculate that this discrepancy may be attributable to the characteristics of our study population, which consisted predominantly of postmenopausal women with low estradiol levels that frequently approached or fell below the detection limit, potentially obscuring its association with MASLD. Future studies in premenopausal women with T2DM are warranted, and the use of more sensitive assays for estradiol may improve the accuracy of clinical evaluations. In addition, whether estrogen replacement therapy during the perimenopausal period may provide protective effects against MASLD remains to be further investigated.

A previous study observed that FSH and LH levels tended to be lower in men with MASLD, whereas progesterone levels showed a positive association with MASLD [28]. In the current study, although progesterone was positively correlated with CAP in men and with LSM in women among patients who underwent FibroScan assessment, no overall association between progesterone and MASLD was observed in univariate logistic regression. This discrepancy may be attributable to our larger sample size (731 participants vs. 432 in the previous study) and differences in the criteria used to define MASLD.

In this study, we developed a logistic regression model incorporating sex hormones, such as testosterone and estradiol, and found that the sex hormone‐based model outperformed most established noninvasive scores, with this finding remaining largely unchanged after internal validation. However, the performance and generalizability of these models require further validation in independent external cohorts before clinical application. Interestingly, in this population, the conventional cutoff values for these noninvasive scores, particularly the FLI (> 60), were insufficient to effectively discriminate between patients with and without MASLD. Although FLI, HSI, and VAI were developed as simple and scalable tools for identifying hepatic steatosis, their diagnostic performance may vary according to the target population [29, 30]. Moreover, the ability of these conventional noninvasive indices to quantify steatosis severity is limited and may be influenced by fibrosis, inflammation, and insulin resistance [29]. Given the distinct clinical and metabolic features of MASLD in patients with T2DM relative to the general MASLD population, future studies should consider patient stratification and the selection of appropriate noninvasive scoring systems for accurate assessment.

Considering the cardiovascular‐renal‐hepatic‐metabolic syndrome, we further analyzed the associations of testosterone and estradiol with cardiovascular event risk and renal function. Due to the limitations of available clinical data, cardiovascular event risk was assessed using a simple scoring system, which provides a reliable estimation of a 10‐year risk for coronary heart disease and ischemic stroke in individuals aged 35–59 years [18]. Most participants fell into the low‐risk category, and no significant differences in sex hormone levels were observed across the risk groups. Elevated APOB/APOA1 ratios have been associated with heightened cardiovascular risk [31]. In our analysis, testosterone levels were inversely associated with the APOB/APOA1 ratio in male patients with T2DM. Correspondingly, a long‐term prospective study reported that testosterone treatment was associated with improved liver function and reduced cardiovascular risk [32]. Among female patients, estradiol levels were positively associated with eGFR. Several limitations should be acknowledged. First, the hospital‐based cross‐sectional design precludes causal inference regarding the associations between exposure factors and MASLD. Second, the multiple exploratory analyses, particularly the correlation analyses, were not formally adjusted for multiple comparisons, which may have increased the risk of Type I error. Third, the use of different imaging modalities for MASLD diagnosis may have introduced potential misclassification bias. Fourth, most female participants were postmenopausal, reflecting the older age distribution commonly observed among patients with T2DM and MASLD in real‐world clinical settings. Nevertheless, our findings remain applicable to the typical T2DM population encountered in clinical practice, although their applicability to premenopausal women requires further validation. Moreover, information regarding the duration and management of hypertension and diabetes, medication use, and lifestyle factors was not available, which may limit the interpretation of our results. Future prospective cohort studies involving larger and more diverse populations, including individuals with and without diabetes and women across different stages of reproductive aging, are warranted to further validate and extend our findings on the role of sex hormones in MASLD diagnosis and risk assessment.

In summary, our study identified sex‐specific associations between circulating sex hormones and MASLD in patients with T2DM. In male patients, higher testosterone levels were inversely associated with MASLD and the APOB/APOA1 ratio. However, among female patients, who were predominantly postmenopausal, no significant associations between sex hormones and MASLD were identified. In addition, estradiol levels were positively associated with eGFR in females. Based on these findings, sex hormone‐based models were developed and showed potential for noninvasive MASLD screening in patients with T2DM.

## Author Contributions

Guixin Li: formal analysis, investigation, software, visualization, writing – original draft. Weiwei Liu: data curation, formal analysis, software, visualization, validation. Ruihua Chen: methodology, project administration. Jinghui Guo: funding acquisition, supervision, writing – review and editing. Long Wang: conceptualization, funding acquisition, resources, supervision, writing – review and editing. Guixin Li and Weiwei Liu have contributed to the work equally and should be regarded as co‐first authors.

## Funding

This study was supported by the Education and Teaching Research Project of the Gastroenterology Sciences Institute, Shanghai Jiao Tong University School of Medicine, JX‐2023‐06‐03, and the Medical Education Research Project of Shanghai Sixth People′s Hospital, Affiliated to Shanghai Jiao Tong University School of Medicine, LYBYHZD202301.

## Disclosure

All authors have read and approved the final version of the manuscript. Long Wang had full access to all of the data in this study and takes complete responsibility for the integrity of the data and the accuracy of the data analysis.

## Ethics Statement

The protocol was approved by the Ethics Committee of Shanghai Sixth People′s Hospital (Approval No. 2020‐069), and written informed consent was obtained from all participants prior to enrollment.

## Conflicts of Interest

The authors declare no conflicts of interest.

## Supporting information


**Supporting Information** Additional supporting information can be found online in the Supporting Information section. Table S1: Correlation of sex hormone levels with CAP and LSM in T2DM patients. Table S2: Univariate logistic regression analysis of MASLD in pre‐ and postmenopausal female T2DM patients. Table S3: Univariate logistic regression analysis of MASLD in T2DM patients stratified by sex and acMASH score. Table S4: Univariate logistic regression analysis of MASLD in T2DM patients stratified by sex and FIB‐4 score. Table S5: AUC comparison of sex hormone‐based model and noninvasive assessment tools for MASLD in male T2DM patients. Table S6: AUC comparison of sex hormone‐based model and noninvasive assessment tools for MASLD in female T2DM patients. Table S7: Bootstrap internal validation of the sex hormone‐based models for MASLD screening.

## Data Availability

The data that support the findings of this study are available on request from the corresponding author. The data are not publicly available due to privacy or ethical restrictions.
