# Supplementary material for: Sex Hormone‐Based Models Outperform Conventional Scores for Metabolic Dysfunction‐Associated Steatotic Liver Disease Screening: A Sex‐Specific Analysis in Type 2 Diabetes
Source: J Diabetes Res. 2026 Oct 7;2026:2030936. doi: 10.1155/jdr/2030936 (PMC13643946; doi:10.1155/jdr/2030936)
Supplement: Supplementary file 1 [file JDR-2026-2030936-s001.docx]

|  | Male (n=43) | | | | Female (n=23) | | | |
| --- | --- | --- | --- | --- | --- | --- | --- | --- |
|  | CAP | | LSM | | CAP | | LSM | |
|  | r | P value | r | P value | r | P value | r | P value |
| Testosterone | -0.392 (-0.664, -0.061) | **0.009** | -0.523 (-0.694, -0.271) | **<0.001** | 0.050 (-0.384, 0.496) | 0.819 | 0.338 (-0.037, 0.691) | 0.114 |
| Estradiol | -0.292 (-0.584, 0.048) | 0.057 | -0.254 (-0.529, 0.064) | 0.101 | -0.064 (-0.511, 0.481) | 0.773 | 0.659 (0.350, 0.808) | **0.001** |
| T/E2 | -0.081 (-0.395, 0.270) | 0.604 | -0.294 (-0.555, 0.011) | 0.056 | 0.151 (-0.361, 0.550) | 0.491 | -0.081 (-0.526, 0.343) | 0.713 |
| FSH | -0.113 (-0.422, 0.235) | 0.470 | 0.003 (-0.278, 0.306) | 0.984 | 0.014 (-0.504, 0.437) | 0.948 | -0.317 (-0.682, 0.172) | 0.141 |
| LH | -0.146 (-0.453, 0.178) | 0.350 | 0.016 (-0.310, 0.361) | 0.921 | -0.067 (-0.546, 0.400) | 0.761 | -0.286 (-0.662, 0.188) | 0.185 |
| DHEA | 0.265 (0.006, 0.523) | 0.086 | 0.316 (0.009, 0.586) | **0.039** | 0.078 (-0.397, 0.547) | 0.723 | 0.381 (-0.022, 0.684) | 0.073 |
| Progesterone | 0.303 (-0.002, 0.571) | **0.048** | 0.269 (0.002, 0.487) | 0.081 | 0.007 (-0.407, 0.439) | 0.974 | 0.458 (0.071, 0.774) | **0.028** |
| Prolactin | 0.137 (-0.175, 0.389) | 0.377 | 0.044 (-0.268, 0.362) | 0.779 | -0.228 (-0.635, 0.212) | 0.295 | -0.039 (-0.483, 0.405) | 0.861 |

**Table S1. Correlation of sex hormone levels with CAP and LSM in T2DM patients**

Data are expressed as r (95% CI), with confidence intervals derived from 1,000 bootstrap resamples.

Abbreviations: T/E2, Testosterone-to-estradiol ratio; FSH, Follicle-stimulating hormone; LH, Luteinizing hormone; DHEA, Dehydroepiandrosterone.

**Table S2. Univariate logistic regression analysis of MASLD in pre**- **and postmenopausal female T2DM patients**

|  | **Premenopausal women** | | **Postmenopausal women** | |
| --- | --- | --- | --- | --- |
|  | OR (95%CI) | P value | OR (95%CI) | P value |
| Testosterone | 2.739 (0.434, 17.295) | 0.284 | 1.528 (0.655, 3.565) | 0.327 |
| Estradiol | 1.001 (0.998, 1.004) | 0.641 | 0.999 (0.996, 1.002) | 0.396 |
| T/E2 | 0.957 (0.002, 429.137) | 0.989 | 1.658 (0.225, 12.230) | 0.620 |
| FSH | 0.984 (0.960, 1.008) | 0.184 | 0.997 (0.984, 1.011) | 0.677 |
| LH | 0.985 (0.930, 1.042) | 0.592 | 0.995 (0.973, 1.018) | 0.692 |
| DHEA | 1.006 (0.998, 1.013) | 0.143 | 1.003 (0.999, 1.007) | 0.169 |
| Progesterone | 1.017 (0.953, 1.085) | 0.610 | 0.378 (0.064, 2.225) | 0.282 |
| Prolactin | 0.998 (0.995, 1.001) | 0.157 | 0.997 (0.994, 1.000) | **0.020** |

Abbreviations: T/E2, Testosterone-to-estradiol ratio; FSH, Follicle-stimulating hormone; LH, Luteinizing hormone; DHEA, Dehydroepiandrosterone.

|  | **Male** | | | | **Female** | | | |
| --- | --- | --- | --- | --- | --- | --- | --- | --- |
|  | **acMASH<4.15 (n=433)** | | **acMASH≥4.15 (n=56)** | | **acMASH<4.15 (n=172)** | | **acMASH≥4.15 (n=70)** | |
|  | OR (95%CI) | P value | OR (95%CI) | P value | OR (95%CI) | P value | OR (95%CI) | P value |
| Testosterone | 0.917 (0.882, 0.954) | **<0.001** | 0.786 (0.664, 0.930) | **0.005** | 1.202 (0.510, 2.834) | 0.674 | 6.474 (0.847, 49.481) | 0.072 |
| Estradiol | 1.001 (0.996, 1.006) | 0.644 | 1.000 (0.981, 1.020) | 0.980 | 0.999 (0.996, 1.001) | 0.342 | 1.001 (0.998, 1.005) | 0.470 |
| T/E2 | 0.692 (0.574, 0.833) | **<0.001** | 0.672 (0.433, 1.043) | 0.077 | 0.982 (0.117, 8.214) | 0.987 | 4.107 (0.080, 210.700) | 0.482 |
| FSH | 0.971 (0.946, 0.998) | **0.033** | 0.915 (0.754, 1.111) | 0.370 | 1.003 (0.990, 1.015) | 0.677 | 0.980 (0.959, 1.000) | 0.054 |
| LH | 0.953 (0.909, 0.998) | **0.042** | 0.940 (0.758, 1.165) | 0.574 | 1.004 (0.983, 1.026) | 0.718 | 0.972 (0.930, 1.016) | 0.206 |
| DHEA | 1.002 (1.000, 1.004) | **0.043** | 1.011 (1.001, 1.020) | **0.024** | 1.003 (0.999, 1.007) | 0.164 | 1.007 (0.999, 1.015) | 0.098 |
| Progesterone | 0.766 (0.302, 1.944) | 0.575 | 0.588 (0.066, 5.233) | 0.634 | 1.030 (0.949, 1.117) | 0.481 | 0.989 (0.908, 1.078) | 0.808 |
| Prolactin | 1.000 (0.999, 1.002) | 0.756 | 0.997 (0.988, 1.006) | 0.499 | 0.998 (0.996, 1.000) | 0.085 | 0.995 (0.992, 0.999) | 0.022 |

**Table S3. Univariate logistic regression analysis of MASLD in T2DM patients stratified by sex and acMASH score**

**Table S4. Univariate logistic regression analysis of MASLD in T2DM patients stratified by sex and FIB-4 score**

|  | **Male** | | | | **Female** | | | |
| --- | --- | --- | --- | --- | --- | --- | --- | --- |
|  | **FIB-4<1.3 (n=287)** | | **FIB-4≥1.3 (n=202)** | | **FIB-4<1.3 (n=140)** | | **FIB-4≥1.3 (n=102)** | |
|  | OR (95%CI) | P value | OR (95%CI) | P value | OR (95%CI) | P value | OR (95%CI) | P value |
| Testosterone | 0.897 (0.850, 0.947) | **<0.001** | 0.915 (0.867, 0.965) | **0.001** | 3.224 (1.003, 10.356) | **0.049** | 0.874 (0.307, 2.484) | 0.800 |
| Estradiol | 1.001 (0.994, 1.008) | 0.773 | 1.003 (0.996, 1.011) | 0.377 | 1.000 (0.998, 1.002) | 0.726 | 0.999 (0.993, 1.004) | 0.562 |
| T/E2 | 0.669 (0.533, 0.841) | **0.001** | 0.686 (0.524, 0.900) | **0.006** | 13.878 (0.521, 369.453) | 0.116 | 0.447 (0.038, 5.332) | 0.525 |
| FSH | 0.957 (0.918, 0.997) | **0.036** | 0.977 (0.943, 1.012) | 0.194 | 1.003 (0.990, 1.016) | 0.663 | 0.985 (0.966, 1.005) | 0.149 |
| LH | 0.928 (0.862, 0.998) | **0.044** | 0.973 (0.919, 1.030) | 0.344 | 0.998 (0.976, 1.020) | 0.827 | 0.992 (0.955, 1.031) | 0.699 |
| DHEA | 1.004 (1.001, 1.006) | **0.002** | 1.000 (0.997, 1.003) | 0.967 | 1.003 (0.999, 1.008) | 0.166 | 1.002 (0.995, 1.009) | 0.569 |
| Progesterone | 0.882 (0.326, 2.384) | 0.805 | 0.468 (0.093, 2.367) | 0.359 | 0.998 (0.939, 1.061) | 0.957 | 1.149 (0.712, 1.853) | 0.570 |
| Prolactin | 1.000 (0.998, 1.002) | 0.872 | 1.000 (0.998, 1.002) | 0.854 | 0.997 (0.995, 0.999) | **0.010** | 0.998 (0.994, 1.002) | 0.282 |

**Table S5. AUC comparison of sex hormone**-**based model and noninvasive assessment tools for MASLD in male T2DM patients**

|  | AUC (95% CI) | Cutoff value | P value |
| --- | --- | --- | --- |
| Sex hormone-based model | 0.820 (0.784, 0.857) | 0.644 | - |
| FLI | 0.743 (0.699, 0.787) | 98.223 | **0.0004** |
| HSI | 0.794 (0.754, 0.833) | 35.928 | **0.0264** |
| LAP | 0.796 (0.755, 0.836) | 33.450 | 0.1643 |
| VAI | 0.745 (0.701, 0.789) | 1.715 | **0.0004** |

Sex hormone-based model for males: Logit (Probability of MASLD) =−7.466 −0.022×Age +0.313×BMI +0.357×Smoking (No: 0; Yes: 1) +0.018×Hypertension (No: 0; Yes: 1) +0.237×TC −0.719×HDL-C +0.079×HbA1c +0.029×ALT −0.049×Testosterone +0.002×Estradiol.

P values indicate the statistical significance of the difference in AUC between each noninvasive model and the sex hormone-based logistic regression model, assessed by the DeLong test.

Abbreviations: AUC, Area under the curve; BMI, Body mass index; HDL-C, High-density lipoprotein-cholesterol; HbA1c, Hemoglobin A1c; ALT, alanine aminotransferase; FLI, Fatty liver index; HSI, Hepatic steatosis index; LAP, Lipid accumulation product; VAI, Visceral adiposity index.

**Table S6. AUC comparison of sex hormone**-**based model and noninvasive assessment tools for MASLD in female T2DM patients**

|  | AUC (95% CI) | Cutoff value | P value |
| --- | --- | --- | --- |
| Sex hormone-based model | 0.810 (0.753, 0.867) | 0.578 | - |
| FLI | 0.721 (0.656, 0.786) | 99.013 | **0.0099** |
| HSI | 0.789 (0.730, 0.848) | 35.454 | 0.3603 |
| LAP | 0.755 (0.693, 0.817) | 38.145 | 0.0637 |
| VAI | 0.744 (0.680, 0.808) | 2.356 | **0.0320** |

Sex hormone-based model for females: Logit (Probability of MASLD) = −3.962 +0.001×Age +0.219×BMI +1.578×Smoking (No: 0; Yes: 1) −0.023×Hypertension (No: 0; Yes: 1) +0.181×TC −1.971×HDL-C −0.044×HbA1c +0.042×ALT +0.171×Testosterone −0.002×Estradiol.

P values indicate the statistical significance of the difference in AUC between each noninvasive model and the sex hormone-based logistic regression model, assessed by the DeLong test.

Abbreviations: AUC, Area under the curve; BMI, Body mass index; HDL-C, High-density lipoprotein-cholesterol; HbA1c, Hemoglobin A1c; ALT, alanine aminotransferase; FLI, Fatty liver index; HSI, Hepatic steatosis index; LAP, Lipid accumulation product; VAI, Visceral adiposity index.

**Table S7. Bootstrap internal validation of the sex hormone-based models for MASLD screening**

| Sex | Apparent_AUC | Mean_optimism | Optimism-corrected_AUC (95% CI) |
| --- | --- | --- | --- |
| Male | 0.820 | 0.016 | 0.804 (0.770, 0.843) |
| Female | 0.810 | 0.034 | 0.776 (0.723, 0.829) |

Data are expressed as optimism-corrected AUC (95% CI), with confidence intervals derived from 1,000 bootstrap resamples.
